# How to improve AML outcomes?

**DOI:** 10.1007/s44313-024-00041-7

**Published:** 2024-12-02

**Authors:** Taner Tan, Sinem Civriz Bozdag

**Affiliations:** Department of Hematology, ͣKoc University Medical School, Istanbul, Turkey

**Keywords:** AML, Treatment, Relapsed, Targeted, Mutations, Outcome

## Abstract

Understanding the intricacies of the pathophysiology and genomic landscape has enhanced the long-term outcomes for patients with acute myeloid leukemia (AML). The identification of novel molecular targets has introduced new therapeutic strategies that attempt to surpass the dominance of the "7 + 3 regimen" established in the 1970s. In 2022, the World Health Organization and International Consensus Classification revised their definitions and approaches to AML, reflecting the current and evolving changes at the molecular level. The guidelines are now grounded in a definition of the disease that emphasizes genetic characteristics. Today, we recognize AML as a genetically diverse disease; a retrospective study identified 5234 driver mutations across 76 genes or genomic regions, with two or more drivers observed in 86% of patients (Papaemmanuil et al., N Engl J Med 374:2209–21, 2016).

Integration of these genomic data resulted in changes in the blast thresholds for diagnosis. According to the WHO criteria, there is no minimum threshold for AML with genetic abnormalities (with the exception of 20% required for AML with BCR:ABL1 and AML with CEBPAbZIP mutations). Conversely, AML is defined by ICC as “at least 10% blasts in the bone marrow or peripheral blood with recurrent genetic abnormalities (with the exception of ≥ 20% in AML with BCR::ABL1)." Additionally, ICC announced a new category of MDS/AML with 10%–19% blasts in the bone marrow or peripheral blood. The European Leukemia Network, an esteemed society, updated its risk classification in 2022 based on novel molecular findings, notably emphasizing the significance of measurable residual disease (MRD) in AML treatment strategies [1–4] (Fig. 1).

**Fig. 1:**
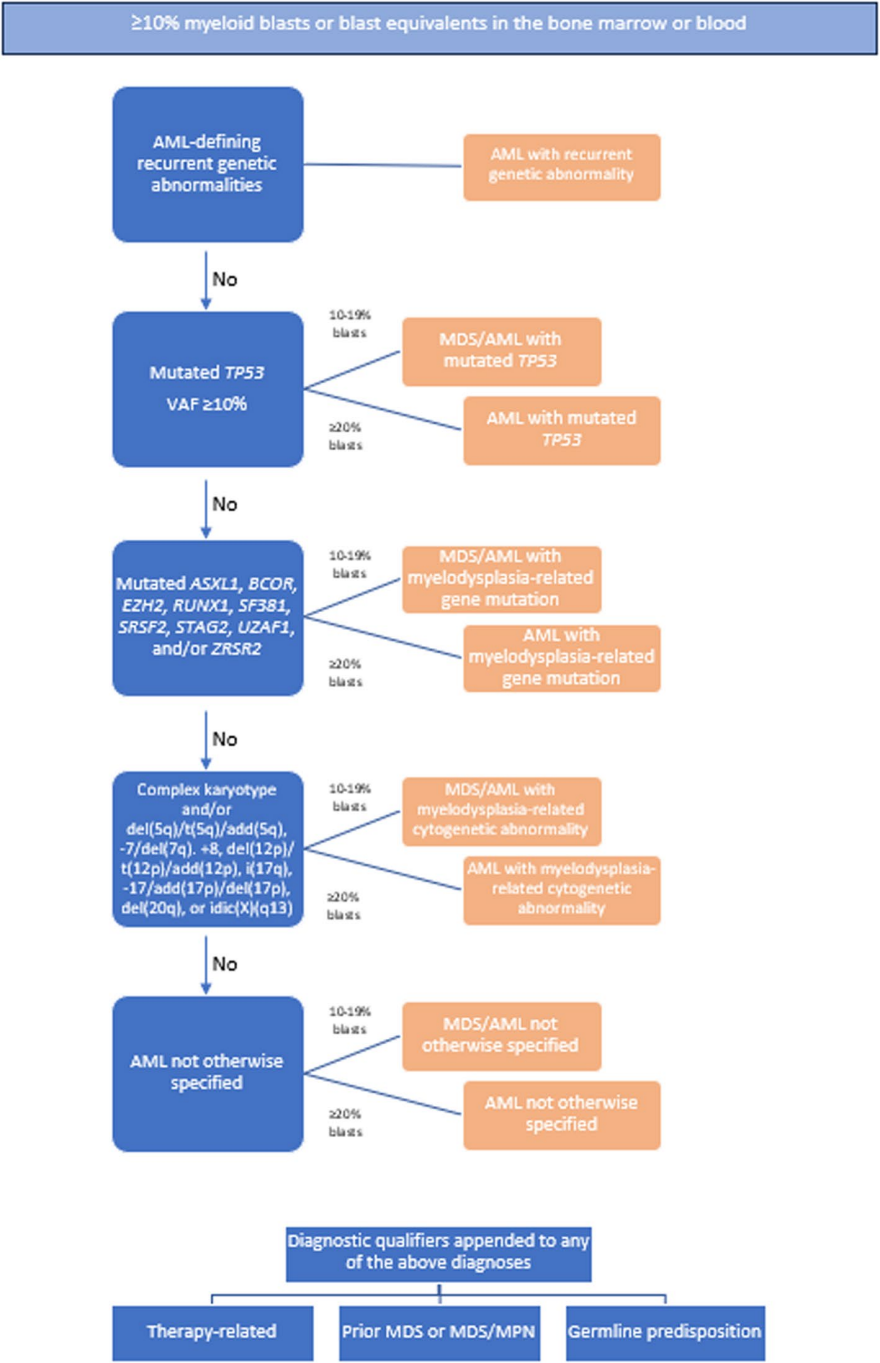
Hierarchical classification of the International Consensus Classification of AML. AML—Acute Myeloid Leukemia, ASXL1—Additional Sex Combs Like 1, BCOR—BCL6 Corepressor, EZH2—Enhancer of Zeste Homolog 2, MDS—Myelodysplastic Syndrome, MPN—Myeloproliferative Neoplasm, RUNX1—Runt-Related Transcription Factor 1, SF3B1—Splicing Factor 3b Subunit 1, SRSF2—Serine/Arginine-Rich Splicing Factor 2, STAG2—Stromal Antigen 2, TP53—Tumor Protein 53, U2AF1—U2 Small Nuclear RNA Auxiliary Factor 1, VAF—Variant Allele Frequency, ZRSR2—Zinc Finger CCCH-Type, RNA-Binding Motif and Serine/Arginine-Rich 2

The most notable changes in the updated recommendations include:

- B-zip in-frame CEPBA mutations, rather than mono- or bi-allelic, are classified within the favorable group.
- FLT3-ITD mutated AML is categorized in the intermediate group, irrespective of the allelic ratio or the presence of an NPM1 mutation.
- The updated risk classification now reflects the importance of ASXL1, BCOR, EZH2, RUNX1, SF3B1, SRSF2, STAG2, U2AF1, and ZRSR2. Pathological variants in one or more of these genes are categorized under AML with myelodysplasia-related gene mutations, defined as an adverse risk.
- NPM1-mutated AML with adverse cytogenetic abnormalities remains an adverse risk.
- Cytogenetic abnormalities, including the MECOM gene and KAT6A::CREBBP, known as disease-defining defects, are now identified as adverse risks (Fig. 2).

**Fig. 2:**
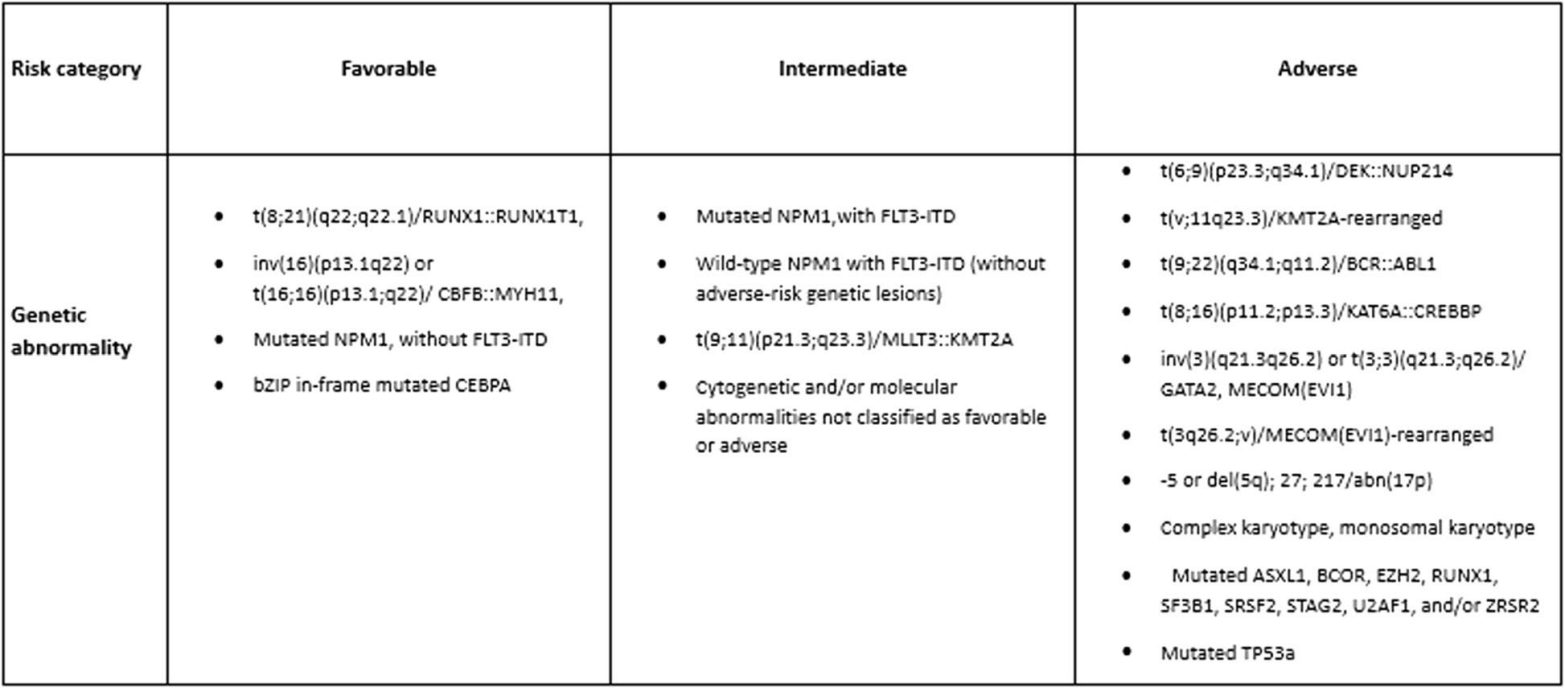
2022 ELN risk classification by genetics at initial diagnosis. AML—Acute Myeloid Leukemia, ASXL1—Additional Sex Combs Like 1, BCOR—BCL6 Corepressor, CEBPA—CCAAT Enhancer Binding Protein Alpha, CREBBP—CREB Binding Protein, EZH2—Enhancer of Zeste Homolog 2, FLT3-ITD—FMS-like Tyrosine Kinase 3-Internal Tandem Duplication, GATA2—GATA Binding Protein 2, KMT2A—Lysine Methyltransferase 2A, MEF2C—Myocyte Enhancer Factor 2C, MLLT3—Myeloid/Lymphoid or Mixed-Lineage Leukemia Translocated to 3, MYH11—Myosin Heavy Chain 11, NPM1—Nucleophosmin 1, NUP214—Nucleoporin 214, RUNX1—Runt-Related Transcription Factor 1, SF3B1—Splicing Factor 3b Subunit 1, SRSF2—Serine/Arginine-Rich Splicing Factor 2, STAG2—Stromal Antigen 2, TP53—Tumor Protein 53, U2AF1—U2 Small Nuclear RNA Auxiliary Factor 1, ZRSR2—Zinc Finger CCCH-Type, RNA-Binding Motif and Serine/Arginine-Rich 2

## Newly diagnosed intensive therapy eligible- FIT AML Patients

### Is there any update for induction therapy?

The "7 + 3" regimen, based on anthracycline and cytarabine, continues to be fundamental, achieving a 60–70% complete remission rate. Importantly, it has been the most effective induction method for these patients. An ideal induction regimen should not only secure high rates of complete remission but also aim for MRD negativity, potentially facilitating subsequent allogeneic hematopoietic stem cell transplantation.

Studies on the doses and types of cytarabine and anthracyclines include the E1900 randomized phase III trial, where a higher dose of anthracycline (daunorubicin, 90 mg/m^2^) conferred a survival benefit predominantly in patients under 50 years, within the favorable and intermediate-risk groups [5]. Subsequent updates revealed that, although increasing the dose from 60 mg/m^2^ to 90 mg/m^2^ did not offer a survival benefit, it led to higher mortality rates [6]. Meanwhile, in the BIG-1 randomized trial, idarubicin was compared with high-dose daunorubicin, revealing greater toxicity in the idarubicin group, with no significant difference in response rates [7].

In terms of cytarabine dosage, the SWOG study demonstrated a notable survival benefit, particularly in younger patients (4-year 52% OS in patients < 45 years), with high-dose cytarabine (HDAC) induction followed by two consolidation cycles. However, these results were comparable to those of the CALGB trial, which employed a standard dose of cytarabine and four cycles of HDAC [8].

scalating the intensity of chemotherapy from 7 + 3 enhanced response rates but also increased toxicity levels. Thus, to enhance outcomes, the addition of a third agent such as an anti-CD33 monoclonal antibody, Flt3 inhibitor, or venetoclax has been explored in young, fit patients. Moreover, in older adult patients with therapy- or MDS-related AML, a 5:1 liposomal cytarabine/anthracycline complex has led to improved outcomes [9, 10].

### Gemtuzumab ozogamycin

(CD33 monoclonal antibody conjugated to calicheamicin) (GO) has demonstrated efficacy in a meta-analysis of five randomized trials, showcasing the benefit of combining GO with standard 7 + 3 chemotherapy in favorable and intermediate-risk ND-AML. The AMLSG09-09, a phase III randomized study, assessed the addition of GO to a regimen of idarubicin, cytarabine, etoposide, and all-trans-retinoic acid in patients with NPM1 positive-AML. Subgroup analysis of this trial revealed a significant EFS improvement in the GO arm among females, patients younger than 70 years, and non-FLT3 (ITD) patients. Furthermore, GO contributed to achieving an MRD-negative state, as determined by reverse transcription polymerase chain reaction [11, 12].

### FLT3 inhibitors

The first-generation FLT3 inhibitor midostaurin, when combined with 7 + 3, has demonstrated significant superiority over 7 + 3 alone, notably reducing the relapse rate [13]. Its effectiveness is evident in both ITD and TKD clones. In the AMLSG 16–10 study, the addition of midostaurin to intensive therapy significantly improved outcomes for both younger and older patients with AML and FLT3-ITD [14]. The beneficial effect of midostaurin persisted in analyses that considered allogeneic HCT as a time-dependent covariate. Due to factors such as mutation acquisition in signaling pathways or the persistence of FLT3-ITD clones, more potent FLT3 inhibitors, like quizartinib, have been explored. In the QUANTUM FIRST trial, the addition of quizartinib to 7 + 3 treatment resulted in a pronounced improvement in OS among patients with ND *FLT3*-ITD mutated AML. Moreover, early and deep clearance of MRD was observed, and the survival benefit was maintained regardless of allogeneic hematopoietic stem cell transplantation [15].

### CPX351

CPX351, a liposomal complex with a 5:1 molar ratio of cytarabine to daunorubicin, has been approved for treating t-AML and AML-MRC in older adult patients [9]. In a retrospective study, the outcomes of older adult patients with AML who received CPX351 versus hypomethylating agent (HMA) + Venetoclax were compared. The OS rates favored the CPX351 group (13 months versus 11 months). Moreover, significantly more patients in the CPX351 group could undergo allogeneic stem cell transplantation. However, these patients also experienced higher rates of hospitalization and infection (including febrile neutropenia) compared to those treated with HMA + Venetoclax [10].

### Venetoclax

Venetoclax induces apoptosis in acute myeloid leukemia by selectively inhibiting pro-apoptotic BCL2. It has been approved in combination with HMA or low-dose cytarabine (LDAC) for patients not fit for induction with intensive chemotherapy aged ≥ 75 years [16]. Following promising results in non-fit patients, studies incorporating intensive chemotherapy with venetoclax in fit patients have been undertaken. Lachowiez et al. assessed the efficacy of venetoclax combined with intensive chemotherapy compared to intensive chemotherapy alone and determined that the combination yields deeper MRD-negative remissions, facilitating transition to allogeneic HSCT in the first remission and enhancing event-free survival [17]. Moreover, Dinardo et al. examined the outcomes in patients with newly diagnosed-AML, employing fludarabine, cytarabine, granulocyte colony-stimulating factor, and ıdarubicin (FLAG-IDA) with venetoclax: composite complete remission (CR) rates reached 89%, with 93% of CR patients achieving MRD negativity, and an estimated 2-year OS of 76% [17]. Additionally, the CAVEAT trial highlighted the advantages of adding venetoclax to the 5 + 2 induction regimen in older adult patients, notably in terms of CR rates. The integration of venetoclax may delay recovery from cytopenia; thus, antimicrobial prophylaxis appears mandatory to mitigate infection rates [18].

### Consolidation; high dose *ARA*-C or less?

Post-remission therapy was defined as cytogenetic and molecular abnormalities. Patients with minimal residual disease-positive favorable-risk or intermediate/adverse-risk groups are candidates for allogeneic hematopoietic stem cell transplantation, either from matched siblings or alternative donors.

The ELN recommends consolidation with intermediate-dose cytarabine (IDAC) (1000–1500 mg/m^2^) alone in non-Flt3 and in combination with Flt3 inhibitors in Flt3 positive AML patients [4]. The National Comprehensive Cancer Network (NCCN), on the other hand, favors high-dose cytarabine, offering it as a choice ranging from 1 to 2 gr/m^2^ for less-fit, intermediate-risk patients and in conjunction with gemtuzumab and daunorubicin for those at favorable risk (NCCN 2023). Moreover, at the American Society of Hematology (ASH) 2023 annual meeting, the randomized BIG-1 trial demonstrated that IDAC, compared with HDAC, serves as the standard for consolidation in patients with non-core binding factor (CBF) AML [7].

### Is there a role for MRD assessment in AML?

**Fig. 3:**
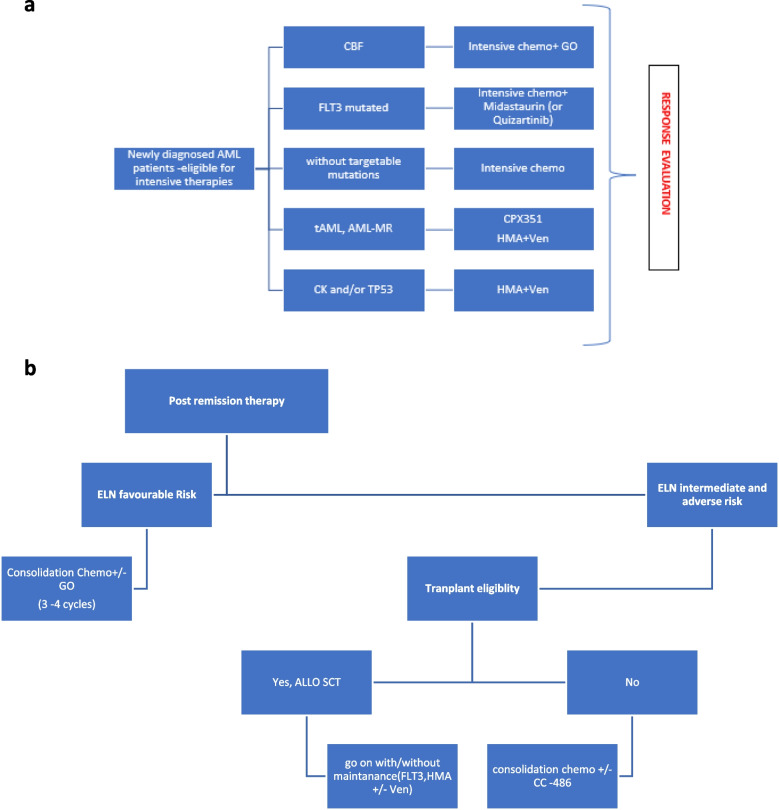
**a** **b** Treatment Algorithm for Newly diagnosed patients with AML fit for intensive therapy. AML—Acute Myeloid Leukemia, CBF—Core Binding Factor, CK—Complex Karyotype, CPX351—Liposomal Daunorubicin and Cytarabine, FLT3—FMS-like Tyrosine Kinase 3, GO—Gemtuzumab Ozogamicin, HMA—Hypomethylating Agents, tAML—Therapy-Related Acute Myeloid Leukemia, AML-MR—Acute Myeloid Leukemia with Myelodysplasia-Related Changes, TP53—Tumor Protein 53, Ven—Venetoclax

MRD can be analyzed using flow cytometry, polymerase chain reaction, or next-generation sequencing in patients with AML. Various studies have demonstrated the prognostic value of MRD on relapse and overall survival [19, 20]. ELN recommendations stipulate timing points as after the second cycle from peripheral blood and at the end of treatment from the bone marrow in CBF and NMP-1 positive AML patients. Furthermore, in all AML groups, assessment with flow cytometry from bone marrow is feasible after the 2nd cycle and at the end of treatment. Additionally, pretransplant MRD serves as a surrogate marker for the long-term outcomes of patients. *DTA* mutations in *DNMT3A*, *TET2*, or *ASXL* can be observed in remission and are unreliable markers of MRD. Notably, a minority of MRD-negative patients may relapse during follow-up, whereas MRD positivity does not necessarily lead to relapse in all patients. Specifically, low-molecular-weight MRD must be interpreted with caution [4, 21] (Fig. 3).

### Non intensive therapy for newly diagnosed

According to the VIALE A study, a combination of HMA and Venetoclax has been widely used in patients not eligible for standard intensive induction chemotherapy [22]. In this study, the venetoclax-azacytidine combination increased survival compared with azacytidine alone. In addition, DiNardo et al. reported promising results using a combination of venetoclax and a 10-day decitabine combination [23].

The response to these non-intensive treatments depends on morphological, cytogenetic, and molecular subgroups. Dinardo et al. identified the molecular patterns of response and treatment failure and highlighted high response rates; durable remission was typically associated with *NPM1* or *IDH2* mutations. Clones that activate signaling pathways, such as FLT3, RAS, or TP53, are responsible for primary and adaptive resistance to venetoclax-based combinations [24]. Unfortunately, remission rates, duration, and unexpected survival rates in TP53-positive patients were lower than those in the non-TP53 group. TP53 positivity can be observed concurrently with complex karyotypes and other mutations, such as *DNMT3A*, *Flt3*, *TET2*, and *RAS*. Variant allele frequencies or single/multi-hit mutations in *TP53* can affect patient outcomes [25].

With the encouraging data on combination therapies, researchers have sought to identify parameters guiding the choice between venetoclax HMA and intensive chemotherapy (IC) for older adult patients. Cherry EM et al. demonstrated that older age, secondary AML, and RUNX1 mutations favor a response to Ven/Aza over IC, whereas the intermediate-risk monocytic AML group showed a preference for IC over Ven/Aza [24]. Furthermore, Konopleva et al. assessed the outcomes in older patients with newly diagnosed AML who were treated with 10-day decitabine plus venetoclax (DEC10-VEN) versus IC. The DEC10-VEN regimen resulted in significantly higher CR/CRi rates, lower relapse rates, and extended overall survival compared to the IC arm [26].

Future studies will focus on reducing the duration of venetoclax and the role of discontinuation of venetoclax, as these are currently continuous treatment schedules. Preliminary data showed the feasibility of 14 days of venetoclax with a similar outcome and less febrile neutropenia and hospitalization [27].

### Can triplets work better than doublets?

In cases involving targeted mutations such as *IDH1/2* or *Flt3*, investigations into doublet/triplet combinations of mutation inhibitors with HMA ± venetoclax treatment have been conducted. A phase III study demonstrated higher CR rates with the combination of gilteritinib and azacytidine compared to azacytidine alone; however, this improvement did not result in a survival advantage for newly diagnosed AML patients [28]. Another phase III study showed that the combination of ivosidenib, azacytidine, and azacytidine offered an event-free survival benefit over azacytidine alone [29].

Furthermore, Short Nj et al. assessed the triplet regimen comprising azacitidine, venetoclax, and gilteritinib in older ND FLT3-mutated AML patients and those of all ages with R/R FLT3-mutated AML, reporting an overall response rate of 67% (CR + CRi + MLFS) and a median overall survival (OS) of 10.5 months [30]. Additionally, Yılmaz et al. explored the efficacy of the triple combination of Quizartinib, Venetoclax (VEN), and decitabine (DAC) in R/R or newly diagnosed FLT3-ITDm AML patients. This regimen proved effective in pretreated and prior FLT3i-exposed R/R FLT3-ITDm patients, achieving a CRc rate of 68% and a median OS of 7.1 months. In frontline treatments, all patients reached CRc [31].

## Salvage regimens for relapsed refractory AML

Mutations should be analyzed in relapsed/refractory patients, irrespective of their status regarding newly diagnosed mutations. Mutations such as *Flt3*, *TP53*, and others in confounding clones or subclones can be observed in relapsed refractory diseases. Using conventional salvage therapies, such as FLAG-Ida and MEC, a CR rate of 40%–50% can be achieved, with approximately one-third of responding patients proceeding to allogeneic stem cell transplantation [32].

Furthermore, the combination of venetoclax with these salvage therapies has deepened the response rate to up to 98%, with 93% MRD negativity, and has increased the transition rate to transplantation to 60% [33].

Gilteritinib is an FLT3 inhibitor that has potent effects by inhibiting both ITD and TKD versions of the enzyme [34]. In the ADMIRAL trial, patients with R/R *FLT3*-mutated AML were randomized to receive gilteritinib monotherapy or salvage chemotherapy. Overall survival was significantly better in the gilteritinib group (9.3 months vs. 5.6 months; HR, 0.64; *p* < 0.001). At the 24-month long-term follow-up, 75% of patients in the gilteritinib arm relapsed, with few relapses occurring after 18 months. Of the 247 patients, 49 were alive for more than 24 months, and 16 received gilteritinib for maintenance in this study [35, 36].

Cortes et al. compared the efficacy of quizartinib, an oral selective type II FLT3 inhibitor, with that of salvage chemotherapy in patients with relapsed or refractory FLT3-ITD acute myeloid leukemia. Notably, the median OS was 6.2 months in the quizartinib group compared to 4.7 months in the chemotherapy group. They concluded that quizartinib offers a survival advantage over salvage chemotherapy and features a more manageable side effect profile, particularly in patients with rapidly proliferating disease and very poor prognosis [37].

### IDH inhibitors (Ivosidenib, olutasidenib, and enasidenib)

Ivosidenib is an IDH1 inhibitor used as monotherapy for the treatment of patients with *IDH1* mutated R/R AML. Ivosidenib as monotherapy could result in CR in 30% of patients with a median response duration of 8 months [30] Lachowiez et al. reported the data from a phase Ib/II study combining ivosidenib with venetoclax ± azacitidine, with response rates of 90% and 83%, respectively [38].

Enasidenib, an IDH2 inhibitor, has been evaluated in patients with *IDH2*-mutated R/R AML. The IDHENTIFY trial assessed patients with *IDH2*-mutated R/R AML treated with enasidenib versus standard of care. Although enasidenib did not improve OS, a post hoc analysis revealed that patients treated with enasidenib, compared to those receiving lower-intensity therapies (LDAC, HMA, or best supportive care, excluding HMA plus venetoclax), experienced improved CR rates and OS (1-year OS 41% vs. 26%) [39].

In conclusion, we can enhance AML outcomes through various strategies, notably by incorporating molecular data, which has revolutionized our perspective on diagnosis and treatment. The updated WHO/ICC classifications have centralised this significant shift, refining our approach. In frontline settings, the use of Flt3 inhibitors in combination with chemotherapy or Ven/HMA, CPX351 in therapy-related and MR AML, and IDH inhibitors with azacitidine has altered our viewpoints and practical methods. The application of Flt-3 and IDH1/2 inhibitors in frontline and salvage settings as doublets or triplets will shape future strategies.

## Data Availability

No datasets were generated or analysed during the current study.
